# HIV healthcare providers’ perceptions on smoking behavior among PLHIV and smoking cessation service provision in HIV clinics in Uganda

**DOI:** 10.18332/tpc/140131

**Published:** 2021-09-11

**Authors:** Kellen Nyamurungi Namusisi, Frances Thirlway, Noreen D. Mdege, Joseph K. B. Matovu

**Affiliations:** 1 Department of Health Policy Planning and Management, School of Public Health, Makerere University, Kampala, Uganda; 2 Department of Sociology, University of York, York, United Kingdom; 3 Department of Health Sciences, University of York, York, United Kingdom; 4 Department of Disease Control and Environmental Health, School of Public Health, Makerere University, Kampala, Uganda; 5 Department of Community and Public Health, Busitema University Faculty of Health Sciences, Mbale, Uganda

**Keywords:** HIV, care providers, smoking behavior, cessation services, integration support, Uganda

## Abstract

**INTRODUCTION:**

Integration of smoking cessation interventions into HIV care can play a crucial role in reducing the growing burden of disease due to smoking among people living with HIV (PLHIV). However, there is a dearth of information on HIV care providers’ perspectives towards integrating smoking cessation interventions into HIV care programs. We explored HIV healthcare providers’ perceptions on the smoking behavior among PLHIV, and the provision of smoking cessation services to PLHIV who smoke within HIV care services in Uganda.

**METHODS:**

Semi-structured face-to-face qualitative interviews were conducted with 12 HIV care providers between October and November 2019. Data were collected on perceptions on smoking among HIV-positive patients enrolled in HIV care, support provided to PLHIV who smoke to quit and integrating smoking cessation services into HIV care programs. Data were analyzed deductively following a thematic framework approach.

**RESULTS:**

Findings show that: 1) HIV care providers in HIV clinics had low knowledge on the prevalence and magnitude of smoking among PLHIV who attended the clinics; 2) HIV care providers did not routinely screen HIV-positive patients for smoking and offered sub-optimal smoking cessation services; and 3) HIV care providers had a positive attitude towards integration of tobacco smoking cessation services into HIV care programs but called for support in form of guidelines, capacity building and strengthening of data collection and use as part of the integration process.

**CONCLUSIONS:**

Our study shows that HIV care providers did not routinely screen for tobacco use among PLHIV and offered suboptimal cessation support to smoking patients, but had a positive attitude towards the integration of tobacco smoking into HIV care programs. These findings suggest a favorable ground for integrating tobacco smoking cessation interventions into HIV care programs.

## INTRODUCTION

Tobacco use is one of the greatest epidemics in the world killing about 8 million people annually^1^. Around 80% of the 1.1 billion smokers worldwide live in low- and middle-income countries, where the burden of tobacco-related illness and death is heaviest^1^. In Sub-Saharan Africa, studies show high smoking rates among people living with HIV (PLHIV)^2,3^. For example, for Uganda, the prevalence of smoking among men living with HIV is two-fold that among general population men (20% vs 10%), and threefold in women living with HIV when compared to general population women (6% vs 2%)^4^. Smoking is associated with a two-fold increase in mortality in PLHIV^5^. One study reported that up to 24% of AIDS-related deaths were attributable to smoking^6^. Collectively, these findings suggest that smoking is a big public health challenge among PLHIV and call for smoking cessation integration in HIV care services^4^.

Studies have revealed that smoking abstinence among smokers with HIV could improve HIV medication adherence and health-related quality of life, and decrease the risk of smoking-related conditions such as cardiovascular diseases (CVD)^7,8^. Smoking cessation among PLHIV could also decrease the risk of early mortality by 16% and the risk of non-AIDS-defined cancers by 34%^9^.

The Ugandan Ministry of Health adopted the WHO Alcohol, Smoking and Substance Involvement Screening Test (ASSIST) tool to facilitate the identification of smokers and the treatment of tobacco dependence, and made it available to interested professionals^10^. Only a few government primary health facilities and hospitals, particularly those dealing with mental health and other addictions (alcohol, drugs, etc.), provide tobacco-cessation support^10^. The Ministry of Health in Uganda recommends that all PLHIV are screened for smoking, and those who smoke encouraged to quit^11^. The implementation of smoking cessation interventions in primary healthcare largely depends on the perceptions and capabilities of healthcare providers^12-14^. A recent study among PLHIV in Uganda found that brief advice to quit smoking is not consistently offered within HIV care and smoking cessation support for PLHIV who smoke is completely lacking. This is despite the potential of smoking cessation to improve multiple health outcomes in this population^15^.

## METHODS

### Study design

This study was a cross-sectional, qualitative study in which interviews were conducted with HIV healthcare providers. It was carried out alongside a survey of HIV clinic participants on tobacco use^4^ and a qualitative study with PLHIV^16^.

### Study site

The study was conducted in 10 districts of Uganda (Arua, Gulu, Hoima, Jinja, Kampala, Kabarole, Lira, Mbarara, Moroto, and Wakiso) which were selected based on their high HIV and tobacco use prevalence, or the fact that they were tobacco growing districts^17^. In each of the 10 districts, we used information on ART-accredited health facilities from Uganda’s HIV treatment guidelines^18^ and information from District Health Officials to select two high patient volume HIV clinics from which study participants were recruited. The selection of the health facilities ensured the inclusion of HIV care facilities from different levels of the healthcare system in Uganda, including the national referral hospital, regional referral hospital, district hospital, Health Centre IV, and Health Centre III^18^.

### Study population and participants’ selection

We interviewed twelve healthcare providers caring for PLHIV in selected HIV care clinics. Healthcare providers were identified with the help of the overall health facility management and the HIV clinic manager/officer-in-charge, after explaining the purpose of the study.

### Data collection procedure

An interview guide with open-ended questions was developed and all interviewers were involved in a training day to agree how this would be used. The guide included exploratory questions on: smoking behaviors among the clientele of the HIV clinics, the nature of smoking cessation services offered in HIV clinics, barriers and facilitators of smoking cessation, healthcare providers’ knowledge and skills on smoking cessation integration, perceptions on smoking cessation integration and support required (Table 1). Semi-structured face-to-face interviews were conducted between October and November 2019. All interviews were conducted in English by six trained researchers with over five years’ experience in qualitative data collection. The two first interviews served as a pilot in terms of ensuring the topic guide was clear, and the research team stayed in-touch with interviewers in the field via a WhatsApp group. All interviews were audio-recorded, and additional notes were taken during the interview. Written consent was obtained from participants. Interviews took between 1–1.5 hours.

**Table 1:** HIV and tobacco prevalence in study regions and districts

*Region*	*District*	*Current tobacco use prevalence* *^19^* *%*	*HIV prevalence at regional level* *^14^* *%*	*Other characteristics* *^17^*
West Nile	Arua	14.5	3.1	Tobacco growing region
Bunyoro Tooro	Hoima	5.2	5.7	Tobacco growing region
	Kabarole	6.3		
Acholi Lango	Gulu	8.3	7.2	
	Lira	6.8		
Busoga	Jinja	2.0	5.1	
Central 1	Kampala	3.2	6.9	
Ankole	Mbarara	7.0	7.9	
North East	Moroto	24.8	3.7	Tobacco growing region
Central 1	Wakiso	2.9	7.6	

### Data analysis

All interviews were transcribed verbatim and the transcribed data were anonymized and saved on a password-protected computer which was only accessed by the study team. Data analysis was mainly done using a deductive approach based on the topics in the interview guide, although there were some themes that emerged during the analysis. KNN initially reviewed five transcripts for completeness and accuracy of transcripts and compared the transcripts with the audio-recordings to ensure consistency. KNN and FT reviewed the transcripts to generate initial codes that were used in coding the rest of the interviews. Based on the codes that were generated, KNN and JBKM read through the transcripts, applying the codes to relevant text as needed. We summarized data in a spreadsheet, which enabled us to compare experiences by reading down each thematic column, whilst preserving each narrative as a unit by reading along each row^20^.

Similar units were grouped together to form sub-themes. These included: 1) How HIV services were organized and tobacco cessation, 2) Existing linkages/partnerships with HIV clinics, 3) Existence and nature of HIV-Tobacco cessation integration or advice, 4) Characteristics of PLHIV who smoke, 5) HW knowledge on tobacco use among PLHIV, 6) Capacity of HIV clinics to offer tobacco cessation services, 6) Factors/why PLHIV smoke, 7) Knowledge on effects of smoking on PLHIV, and 8) Factors that would help or hinder tobacco cessation integration into HIV services/challenges. Sub-themes were then grouped into the following three themes that were determined *a priori*: 1) Perceptions on smoking behavior among PLHIV, 2) Nature of cessation support offered to PLHIV who are in HIV care, and 3) Support required for smoking cessation integration in HIV care. For each sub-theme and theme, we obtained relevant quotations for use in presenting the results.

## RESULTS

### Participant characteristics

Of the 12 healthcare providers interviewed two were doctors, four were clinical officers, five were nurses and one was a counsellor. Participants included seven women and five men. The average number of years worked in the HIV clinics was 4.5 years; the minimum and maximum were one and twelve years, respectively. The majority (86%) of the HIV care providers were the officer-in-charge of the ART clinics in the health facilities visited. The main results are detailed in the sub-sections below.

### Participant perceptions of smoking behavior among PLHIV

Our study focused on the use of combusted tobacco (i.e. either manufactured or self-rolled cigarettes or pipe). In general, most healthcare providers reported that they had low knowledge on prevalence and magnitude of tobacco use among PLHIV, except in Karamoja region where snuff use was reported as the norm for both men and women and in northern Uganda (Lira and Gulu), where smoking was reported to be common among PLHIV. In Arua West Nile, healthcare workers said the chances of PLHIV using tobacco are very high although they would not easily disclose this to the healthcare providers. Non-disclosure of smoking behavior among PLHIV was mainly attributed to stigma, fear of healthcare worker reaction and inadequate skills among the healthcare providers to engage PLHIV on smoking:

*‘Some of them open up but some of them don't depending on the way you have interacted with them, some don't open up but some open up and say “oh, I use this kind of stuff” that all depends on your skills.’ (Healthcare provider, Eastern Region)*

According to healthcare providers, smoking behavior was much more common among male than female HIV patients, and this was true regardless of age and area of residence (rural vs urban). Smoking was also perceived to be more common among patients in lower socioeconomic status groups who were in occupations such as casual laborers, security guards, commercial sex workers, fishermen, and those who were unemployed.

### Participant perceptions of the nature of smoking cessation services offered to PLHIV who smoke

In terms of provision of smoking cessation advice and support to smoking patients in HIV care, some of the healthcare providers reported that they screened for smoking behavior and offered brief advice to smokers to reduce or quit smoking. In a few cases, healthcare providers said they did not screen for tobacco use but screened for other behaviors such as alcohol use. In situations where screening and advice on smoking cessation was offered, the advice was mainly linked to effects of smoking on ART treatment outcomes such as drug interactions, namely the potential reduction of the efficacy of ART treatment; poor adherence; and weakened immunity which increases susceptibility to infections such as TB:

*‘One most advice that we give, and we let them know is you know the tobacco you are smoking: one, it may have an effect on the drugs you are taking. The effect could be either it can reduce the efficacy of the drugs. Two, it can react and form more toxic side effect to you as per drug swallower where you have problems. Three, with your smoking at times you may say okay since I am smoking I don't need to take the drugs because it may have problems you will refuse to take your drugs rightly because you enjoy smoking and therefore adherence becomes a problem.’ (Healthcare provider, West Nile Region)*

Most of the healthcare providers reported that PLHIV in care who smoked found it hard to stop smoking, because of the perceived positive effect of smoking on stress:

*‘… it's quite hard on their part [to quit smoking] because these problems add on their stress and as I said, most people report that a cigarette calms them down when they are stressed. For example, most of our clients are not doing well financially yet they have loads of responsibilities and problems to solve, so at times it becomes hard for them to stop the addiction because of such problems.’ (Healthcare provider, Western Region).*

Some healthcare providers mentioned that a few patients stop using tobacco when they are diagnosed with HIV while others cut down and eventually quit as a result of constant reminders during health education sessions on the dangers of tobacco use. There was a perception that immediate quitting was difficult among PLHIV and cutting down slowly until they quit was more practical, as one healthcare provider from the West Nile region highlighted:

*‘… as they smoke, I want to be frank, some they will tell you here, you can tell them the side effects of cigarettes smoking but practical they will say no, doctor I can't leave it overnight. You do it slowly, they will go and do the same practice at home. They do it at home …’ (Healthcare provider, West Nile Region)*

### Participant perceptions of the support required for smoking cessation integration in HIV care

Most participants felt that integration of smoking cessation services into HIV care programs was crucial to improve the health outcomes of HIV patients enrolled in HIV care. However, they thought that the current level of integration of smoking cessation services into HIV care was largely sub-optimal and not routinely provided, as illustrated in the quotations below:

*‘There is need for specialized training for health workers at the forefront of this battle to enable them function better than they are currently ... from a public health point of view, the authorities and bodies concerned have not yet done much ... There is need to introduce and support clinics with electronic medical records systems.’ (Healthcare provider, Central Region)*

*‘If it is a brought up as an awareness that you have to talk to these people, we can integrate this and we make sure to create awareness to all health workers. but most of them don't record them ... that information is not documented in most cases save for a few.’ ( Healthcare provider, Central Region )*

When asked how tobacco smoking cessation integration into HIV care programs can be strengthened, all the three categories of study participants mentioned health system strengthening including capacity building among healthcare providers on delivering smoking cessation interventions, establishment of smoking cessation guidelines, improved smoking behavior data collection, collation and use, and provision of information, education and communication (IEC) materials on smoking cessation integration, as crucial for supporting the integration of smoking cessation interventions in HIV care, as shown below.

#### Build healthcare providers’ capacity in smoking cessation integration into HIV care

In general, while all healthcare providers were motivated and supportive of smoking cessation integration into HIV care, insufficient training was perceived as the biggest barrier to smoking cessation integration. Healthcare providers also reported lack of confidence to follow up on clients who smoke and support them to quit. Based on this, healthcare providers recommended a comprehensive capacity building program on smoking cessation integration in HIV care for all the HIV clinic staff, as summarized in the quotation below:

*‘All staff should be trained on ways to directly stop smoking, like which ways to directly stop smoking. If there is a possibility of providing a hand by linking former smokers to current smokers to provide testimonies, it would be helpful. We can advise that stop but the former smoker can have specific advices to give. People have reasons for smoking, i.e. me I started to smoke because my parents refused to take me to school and the sight of my former classmates going / continuing with school frustrated me. Others will say that me I got frustrated with the condition and opted for tobacco …’ (Healthcare provider, Northern Region)*

#### Develop and make available smoking cessation guidelines

Participants highlighted the need for smoking cessation guidelines or manuals to facilitate *integration:*

*‘We can have those brochures, we can have some guidelines to help on that with very clear information where someone can easily read and get a message and pass it to the client and the same message.’ (Healthcare provider, Western Region)*

Similarly, materials such as flip charts, instructional cards, and posters for health workers, were recommended to facilitate effective counselling sessions. One respondent suggested that integration of smoking cessation into HIV care should come with an increased supply of smoking cessation pharmacotherapies, as the following quotation illustrates:

*‘Maybe if they can provide with therapies that can help people to stop smoking, like the drugs; also the ART clinics be given the medicines to be used in smoking cessation.’ (Healthcare provider, Karamoja Region)*

Others suggested smoking-specific programs, as with other health conditions, to support health workers in integrating smoking cessation into HIV care.

*‘We need guidelines like it is on TB and HIV, we should also be provided with guidelines on smoking.’ ( Healthcare provider, Central Region )*

#### Strengthen smoking cessation data management and use

Some healthcare providers mentioned that information on smoking behavior among ART clients was recorded on a blue card and in some clinics on the patients’ files. We asked to look at the smoking data that are collected but we could not trace any records in any of the facilities. Discussions with HIV care providers further revealed that the data on smoking behavior, if collected, are neither used beyond collection nor submitted to the Ministry of Health through the existing national health management information system. With these challenges, respondents suggested that any smoking cessation integration program should strengthen data collection (by including tobacco smoking on the routine assessment forms), storage, collation, and use:

*‘Clinics should be oriented and supported in the use of the data captured to plan for services better.’ ( Healthcare provider, Central Region )*

### Provide information education and communication (IEC) materials on smoking cessation integration in HIV care

Healthcare providers noted that the use of IEC materials improves the quality of health education and counselling and generates a standard message to all the HIV clients who smoke.

Healthcare providers requested IEC materials both visual and audio such as patient charts, demonstration materials such as leaflets, brochures as well as a video program that would, for example, be used to communicate the health effects of smoking on the human body:

*‘Like we can have those brochures, we can have some guidelines to help on that with very clear information where someone can easily read and get a message and pass it to the client and the same message. Because the same message in our hospital should be the same message in a health center II, because you cannot know where the client is going … So, we have those materials – IEC materials, we can have some guidelines to support the people.’ (Healthcare provider, Western Region)*

## DISCUSSION

Our study which explored healthcare providers’ perspectives on smoking cessation integration in HIV care in Uganda shows that: 1) HIV healthcare providers had low knowledge on the prevalence and magnitude of tobacco use among PLHIV; 2) screening for tobacco smoking is not routinely done and smoking cessation support is sub-optimal; and 3) healthcare providers viewed integration of smoking cessation into HIV care as essential but there was need for guidance, capacity building, and strengthening data collection and use.

Evidence suggests a high prevalence of tobacco smoking among PLHIV in Uganda^4^. Our finding that HIV care providers had a low knowledge on the prevalence and magnitude of tobacco use among PLHIV could be attributed to the fact that healthcare providers do not routinely screen for, or ask about, tobacco smoking. This is consistent with evidence from previous studies which suggests that HIV care providers may fail to identify patients who are current smokers during the history-taking and physical examination^13,9^. A study that assessed awareness of current smoking among HIV and non-HIV healthcare providers in the US, found that HIV healthcare providers were less likely to recognize current smoking in patients reporting bothersome signs and symptoms than non-HIV healthcare providers^13^.

In our study, healthcare providers indicated that HIV patients who smoke tended to hide their smoking habits for fear of being blamed for continuing to smoke against medical advice. Our findings are consistent with studies which have found widespread under-reporting of tobacco use in the general population^21^ and among PLHIV in SSA, particularly by women^21^. These findings suggest the need to equip healthcare providers with skills to identify smokers and engage them in ways that encourage open and supportive discussions on the issue.

We found that smoking cessation support was not routinely provided, which is consistent with previous studies that indicated that few healthcare professionals participate in the full range of activities needed to support patients to quit smoking tobacco^22,23^. For example, a study in primary healthcare settings in the US found that only 23.7% of healthcare professionals reported assessing the willingness of the patients to quit smoking; 17.9% reported discussing counselling options with smokers; and 6% reported preparing their patients for withdrawal symptoms^21^. This suggests a need to support healthcare providers to provide a full range of smoking cessation services, from asking the patients about their smoking status, offering advice and support, to arranging follow-up to evaluate their progress^22^. This support could include clear intervention guidelines and manuals, and information, education and communication (IEC) materials to use in facilitating tobacco smoking cessation counselling sessions.

Our findings that the majority of the healthcare providers reported that they lacked the skills needed to implement effective smoking cessation interventions are similar to findings from other studies^13,23,24^. A US online survey^13^ found that only 22.9% of healthcare providers surveyed reported that they had ever received formal tobacco treatment training. Another study of Canadian midwives’ experiences of approaching women smokers in antenatal care^25^ found that healthcare providers lacked skills on how to open the issue of smoking cessation with the patients, how to follow up these initial discussions, and how to deliver information in a way that could be well received by the smokers. A systematic review by Carson et al.^26^, demonstrates that training of healthcare providers in how to provide effective smoking cessation can increase the number of health providers who can correctly identify smokers, provide the necessary smoking cessation counselling, help smokers to set a quit date, and provide follow-up appointments to those intending to quit smoking. It is also crucial for improving quit rates.

### Strengths and limitations

This study had limitations and strengths. Given its qualitative nature, the findings presented can only relate to what the interviewed participants think about smoking cessation integration into HIV care and cannot be generalized to all the health professionals in Uganda. The number of participants is relatively small, and they had varied backgrounds including medicine, nursing, and counselling. However, our findings do allow us to build hypotheses regarding the challenges inherent in achieving full smoking cessation integration in HIV care within the settings studied. The strengths of our study include the fact that our study findings are based on data from twelve health facilities across all four regions of Uganda. These findings provide valuable insights into current healthcare practices as they pertain to the integration of smoking cessation into HIV care, and these insights can help to inform the integration of smoking cessation interventions into HIV care programs in Uganda. To the best of our knowledge, this is the first study to explore healthcare providers’ perceptions on smoking cessation integration in HIV care in Uganda.

## CONCLUSIONS

Although healthcare providers screen for tobacco smoking among people living with HIV who are in HIV care, this practice is sub-optimal and only guided by individual healthcare provider practices. There are no smoking cessation intervention guidelines, and there is little evidence that smoking individuals receive a full package of interventions to help them go through the quitting process. To achieve full integration of smoking cessation into HIV care, there is a need for training of healthcare professionals in implementing smoking cessation interventions, smoking cessation guidelines and other forms of material support, as well as improved data collection on smoking behaviors and smoking cessation and its use.

## Data Availability

The data supporting this research are available from the authors on reasonable request.
